# Cost analysis of glatiramer acetate vs. fingolimod for the treatment of patients with relapsing-remitting multiple sclerosis in Spain

**DOI:** 10.1186/2191-1991-3-13

**Published:** 2013-05-07

**Authors:** Rainel Sanchez-de la Rosa, Eliazar Sabater, Miguel A Casado

**Affiliations:** 1 Medical & HEOR Department, TEVA Pharmaceutical, Calle Anabel Segura, 11-1ª planta, Alcobendas, Madrid, 28108, Spain; 2 Pharmacoeconomics and Outcomes Research Iberia Edificio CEP Altagracia I Calle Segundo Mata, 1 (2ª Planta), Pozuelo de Alarcón, Madrid, 28224, Spain

**Keywords:** Relapsing-remitting multiple sclerosis (RRMS), Cost analysis model, Economic evaluation, Glatiramer acetate, Fingolimod

## Abstract

**Background:**

Fingolimod is an innovative drug with a significant budget impact in the treatment of MS in Spain. The aim of this study was to calculate the direct cost comparison of glatiramer acetate and fingolimod for the treatment of patients with relapsing-remitting multiple sclerosis (RRMS) in Spain.

**Methods:**

A cost analysis model was developed to compare glatiramer acetate and fingolimod, based on a 1-year time horizon. In addition to the pharmacological costs, resource use was estimated for glatiramer acetate (1 hour of training with nursing staff in self-injection techniques for subcutaneous administration) and fingolimod (vaccination for varicella-zoster virus in 5% of patients, 3 complete blood counts per year, 3 ophthalmology visits for prevention of macular edema, 3 transaminase tests to monitor liver function, and cardiovascular monitoring consisting of 1 ECG before the first fingolimod dose and at 6 hours; 1 day outpatients-hospital visit for cardiological monitoring during 6 hours on the day of the first fingolimod dose, with follow-up of blood pressure and heart rate every hour). The pharmacological costs were calculated based on the ex-factory price of the drugs evaluated, using the doses recommended in the respective Summary of Products Characteristics (SmPC). Total invoicing volume was discounted by 7.5%, as laid down in Spanish Royal Decree 8/2010. Unit costs were obtained from the e-Salud database and the drug catalog. Costs in the model are expressed in €2012.

**Results:**

The cost of annual treatment was €9,439.42 for glatiramer acetate and €19,602.18 for fingolimod, yielding a cost difference of €10,162.76. Assuming a fixed budget of €100,000.00, approximately 10 patients could be treated with glatiramer acetate, compared to 5 with fingolimod.

**Conclusions:**

Fingolimod therapy requires twice the investment as glatiramer acetate.

## Background

Multiple sclerosis (MS) is a chronic, autoimmune, neurodegenerative disease affecting the central nervous system, which is associated with an irreversible progressive disability that causes great concern for the patient. It most affects young adults [1]. The most common clinical form is relapsing-remitting multiple sclerosis (RRMS) which can represent up to 65% of all patients with MS [2]. In Spain, the prevalence reported is from 50–70 cases per 100,000 inhabitants [3,4]. Unfortunately, it is not currently possible to cure MS, the treatments available in the therapeutic armamentarium up to now have centered their action on the anti-inflammatory and disease course-modifying effect, and the purpose of treatment is to prevent recurrence of relapses and accumulation of disability. A few disease-modifying therapies (DMTs) including Interferon beta-1a, beta-1b and glatiramer acetate have been approved for patients with RRMS to delay disease progression and reduce the incidence of relapses [5]. Fingolimod, the first DMT oral formulation, was recently approved by EMA. Therefore, treatment of RRMS has changed with the introduction of fingolimod, whose incremental cost is meaning a significant impact on the budget dedicated to treatment of MS. At this time, it has the highest treatment/cost/patient/year in Spain [6]. Both glatiramer acetate and fingolimod reduce progression and relapses among patients with RRMS. Compared to glatiramer acetate, fingolimod-treated patients were at increased risk of some unintended treatment effects [7,8].

The current financial scenario reinforces the payers’ needs (National Health System, NHS) of review with care the rational use of treatments. According to the spending rule approved by law in 2012 [9] CCAA cannot exceed the reference rate of GDP, representing huge pressure to reduce immediately its budget deficit and direct drug costs. Moreover, the Government of Spain has cut back 2013 Ministry of Health’s budget by 22.6% [10].

This study was carried out with the purpose of evaluating the economic feasibility of management of treatment of RRMS after the introduction of a new drug using a model of direct cost comparison from the Spanish payers’ perspective.

## Methods

### Study design

For the purpose of conducting this study, a cost comparison model was developed and implemented in an application of the Microsoft Excel 2003 software including as therapeutic alternatives glatiramer acetate (GA; Copaxone®, Teva Pharmaceutical Ltd) and fingolimod (Gilenya®, Novartis Europharm Ltd).

### Perspective and time horizon

The perspective of the analysis was that of the payer (NHS, Autonomous Community or hospitals) and the time horizon was set to 1 year, so this is the usual period used by the payer in planning of its budgets.

### Resources

Only resources related to direct health costs that are financed by the health authorities were considered: ***Drugs; Treatment administration***: only for glatiramer acetate [8], for all patients starting treatment was considered necessary to have a one hour session of training by qualified nursing staff, equivalent to a nursing visit; ***Prevention of infections***: only for fingolimod, as recommended in its SmPC [8], was considered vaccination for varicella-zoster virus in 5% of patients and 3 complete blood counts per year; ***Monitoring of liver function***: only for fingolimod, as recommended in its SmPC [8], were considered 3 transaminase tests per year (SGOT, SGPT, GGT); ***Prevention of macular edema***: only for fingolimod, as recommended in its SmPC [8], were considered 3 ophthalmology visits per year; ***Cardiovascular monitoring*:** only for fingolimod, as recommended in its SmPC, was considered an ECG before the first fingolimod dose and at 6 hours. 1 day outpatients-hospital visit for cardiological monitoring during 6 hours on the day of the first fingolimod dose, with follow-up of blood pressure and heart rate every hour [8].

### Costs

The pharmacological costs were calculated based on the ex-factory price of the two drugs evaluated, according to the doses and conditions of use recommended in the respective SmPC. The cost of medical visits is determined as follows. First, the cost of the initial neurologist’s visit is considered. This is based on the yearly cost of patient starting treatment. Then, all successive neurologists’ visits are estimated using the unit cost a typical follow-up visit.

Unit costs of the drugs used in the model were obtained from the e-Salud database [11] and the drug catalog [12]. All costs in the model are expressed in €2012. A 7.5% discount was applied for these prices as laid down in Spanish Royal Decree 8/2010 (of May 20, when extraordinary measures to reduce the public deficit were adopted [13] (Table 1).

**Table 1:** Unit costs

	**Units**	**Cost (€ 2012)**
**Pharmacological cost**		
GA (Copaxone®), 20 mg/mL, 28 1-mL prefilled syringes	Per presentation	€781.25
Fingolimod (Gilenya®) 0.5 mg, 28 hard capsules	Per presentation	€1,600.00
**Visits**		
Cost per hour of nursing care	1 hour	€19.08
Ophthalmology visit	Per visit	€37.88
Day hospital visit	Per visit	€137.59
**Vaccines**		
Varicella vaccine	1 vaccine	€43.50
**Tests**		
Electrocardiogram	Per test	€20.34
Complete blood count	Per test	€6.93
Transaminases	Per test	€4.93
GA: glatiramer acetate.		

### Sensitivity analyses

To reduce the uncertainty of the model assumptions, univariate sensitivity analyses were performed by decreasing values for key parameters in the model. Analyzed parameters included 10% and 20% discount rate in glatiramer acetate or 10% discount in Fingolimod list price.

## Results

The cost of treatment in the first year was €9,439.42 for glatiramer acetate and €19,602.18 for fingolimod, yielding a cost difference of €10,162.76 (Table 2).

**Table 2:** Results of cost analysis of glatiramer acetate versus fingolimod

	**GA**	**Fingolimod**	**GA-Fingolimod**
**Pharmacological cost**	€9,420.34	€19,292.86	***€-9,872.52***
**Administration cost**	€19.08	€0.00	***€19.08***
**Cost of infection management**	€0.00	€22.97	***€-22.97***
**Cost of prevention of macular edema**	€0.00	€113.64	***€-113.64***
**Cost of liver function monitoring**	€0.00	€14.79	***€-14.79***
**Cost of cardiovascular monitoring**	€0.00	€157.93	***€-157.93***
**TOTAL COST PER PATIENT/YEAR**	**€9,439.42**	**€19,602.18**	***€-10,162.76***

*GA*, Glatiramer acetate.

**Figure 1:**
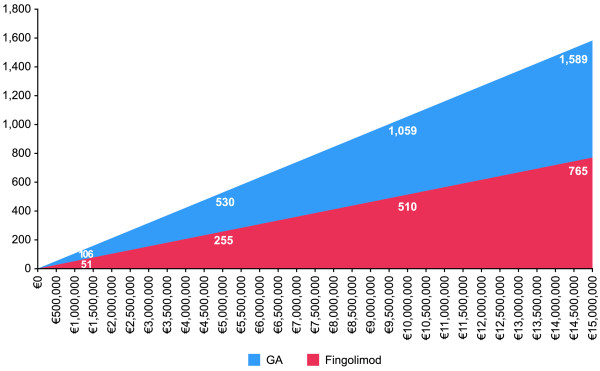
Patients treated with fixed budget.

The ratio of the cost of treatment with fingolimod was 2.1 times the cost of glatiramer acetate, i.e., assuming a fixed budget of €5,000,000.00; approximately 530 patients could be treated with glatiramer acetate and only 255 with fingolimod (Figure 1).

**Figure 2:**
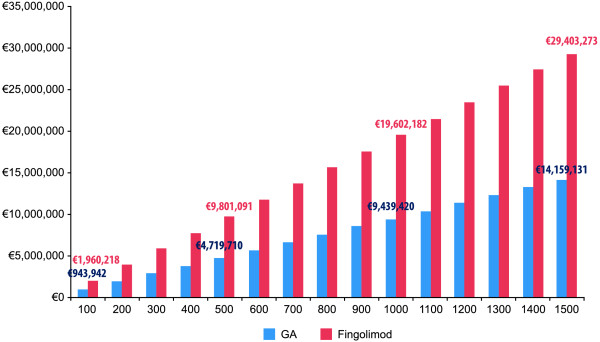
Budget according to number of patients treated.

The cost to treat 1000 patients with glatiramer acetate is €9,439,420, whereas if they were treated with fingolimod the cost would be €19,602,182 (Figure 2). In Table 3 we presented the data from the sensitivity analyses.

**Table 3:** Results of sensitivity analyses

	**GA**	**Fingolimod**	**GA-Fingolimod**
**Base Case**	**€9,439.42**	**€19,602.18**	***€-10,162.76***
**10% rebate in GA & no rebate in Fingolimod list Price**	**€8,478.30**	**€19,602.18**	***€-11,123.80***
**20% rebate in GA & no rebate in Fingolimod list Price**	**€7,536.27**	**€19,602.18**	***€-12,065.91***
**10% rebate in Fingolimod & no rebate in GA list Price**	**€9,439.42**	**€17,672.90**	***€-8,233.48***

*GA*, Glatiramer acetate.

## Discussion

Healthcare decision-making is complex and involves a wide range of factors. For the payer, Spanish NHS, it is very relevant to use a rational criterion when allocating resources, which is based on clinical evidence, efficiency and budget impact of the different therapeutic options available for the management of a given disease [14,15]. In the case of MS, the availability of several drugs for its treatment, each with its particularities and specific profile, makes choosing the most appropriate treatment not easy for physicians, patients, or the payer.

In Spain, MS treatment drugs are dispensed at the hospital pharmacy. Pharmacological direct costs of management of this disabling disease accounts for 52% of all resources required for its management and directly impacts on the cost of each hospital [16]. The average budget impact for the cohort of patients with RRMS under treatment in Spain, considering all the options indicated for first-line treatment, represents an investment of €260 million annually, with an average cost per patient of €11,540 per year [17]. The use of the DMT in real clinical practice setting has been a topic of substantial debate for payers at hospital and CCAA level. In this context, treatment with glatiramer acetate represents a saving of 15.7% over total expenditure. The first oral treatment for this disease, fingolimod, has recently been marketed. This drug is the most expensive one available on the Spanish market, which has led to a substantial incremental cost in the budget dedicated to treatment of MS by the NHS [6].

This study, through an economic model of cost comparison from the perspective of the NHS, provides data on two therapeutic alternatives. The use of glatiramer acetate is a sustainable alternative within the current policy of containment of pharmacological expenditure, without sacrificing efficacy and safety in patients with MS. These results are not new; there are previous studies that have already shown that treatment with glatiramer acetate represents a cost saving of between 5 to 14% as compared to other treatments for MS [18,19].

In Germany, a cost minimization analysis showed that, compared to fingolimod, treatment of MS with interferon beta-1b leads to substantial cost savings from the perspective of society and payer [20]. A Canadian study showed that despite fingolimod having a greater reduction in the annual relapse rate, it has the same net health benefit as treatment with interferon beta-1b [21]. This point should be taken into account together with the greater cost of treatment with fingolimod.

We want to remark that treatment decision it is not only based in cost, which at this time is a key parameter to take into consideration, being particularly important when it is possible to use both treatments according to the patient performance and disease status. Based on costs, it is necessary to point out glatiramer acetate is the best option only in patients with similar health effects, but it could not be the best option in patients with lower health effects. Physicians will always take the treatment decisions taking into consideration the best benefit- risk ratio for the patient.

This study has as a limitation: It has been made from the perspective of the NHS. Therefore, only direct healthcare costs have been taken into account and it did not include the indirect costs related to loss of productivity of patients or caregivers which can represent an important percentage of total cost [22]. In this regard, further studies are needed that can provide differential information on the indirect costs between both therapeutic alternatives.

## Conclusions

Our study provides relevant information for decision making from the perspective of the NHS. In conclusion, treatment of RRMS with fingolimod has twice the impact on the budget than glatiramer acetate. Consequently, treatment of RRMS with glatiramer acetate is an alternative that facilitates sustainability of a universal and comprehensive healthcare system with public funds.

## Abbreviations

GA: Glatiramer acetate (only used in tables/figures); MS: Multiple sclerosis; NHS: National health system; RRMS: Relapsing-remitting multiple sclerosis; SmPC: Summary of product characteristics.

## Competing interests

Teva Pharmaceutical Spain provided funding for this project. E Sabater and MA Casado are employees of Pharmacoeconomics & Outcomes Research Iberia, Madrid, Spain, an independent contract health economic organization that has received research funding from Teva Pharmaceutical. R Sánchez-de la Rosa works for the Medical & HEOR Department of Teva Pharmaceutical.

## Authors’ contributions

RS, ES and MAC conceived the study, participated in its design, contributed to acquisition of the data, interpretation of results and drafting of the manuscript. All authors read and approved the final manuscript.
